# Humanization and Communication Skills: A Cross-Sectional Study in Spanish Nursing Students

**DOI:** 10.3390/nursrep16050171

**Published:** 2026-05-18

**Authors:** Paola Guzmán-De Santa Ana, Alexis Serna-Menor, Ana Martínez-García, Raquel Moreno-Sánchez, Carlos Ruíz-Núñez, Andrés Ignacio García-Notario, Juan Pablo Hervás-Pérez, Ivan Herrera-Peco

**Affiliations:** 1 Unidad de Neurologia y Neurocirugia, Hospital 12 de Octubre, 28041 Madrid, Spain; paola.guzman@salud.madrid.org; 2 Faculty of Pharmacy, Complutense University of Madrid, 28040 Madrid, Spain; alexsern@ucm.es; 3 Pediatric Unit, Hospital Clínica Universitario Lozano Blesa, 50009 Zaragoza, Spain; amartinezgarcia@salud.aragon.es; 4 Facultad de Ciencias de la Salud-HM Hospitales, Universidad Camilo José Cela, Villanueva de la Cañada, 28692 Madrid, Spain; ramoreno@ucjc.edu (R.M.-S.); ivan.herrera@ucjc.edu (I.H.-P.); 5 Instituto de Investigación Sanitaria, HM Hospitales, 28015 Madrid, Spain; 6 Centro de Emergencias Sanitarias 061, Unidad de Innovación, Servicio Andaluz de Salud, 29590 Malaga, Spain; carlos.ruiz.nunez.sspa@juntadeandalucia.es; 7 Facultad de Ciencias Biomédicas y de la Salud, Universidad Alfonso X el Sabio, Villanueva de la Cañada, 28691 Madrid, Spain; apsico@uax.es; 8 Analytical Chemistry Unit, Department of Chemistry in Pharmaceutical Sciences, Faculty of Pharmacy, Complutense University of Madrid, 28040 Madrid, Spain

**Keywords:** communication, emotional intelligence, empathy, humanization of assistance, nursing students

## Abstract

**Introduction:** Humanized care is a core indicator of nursing quality, yet its prevalence and determinants among Spanish undergraduates remain unclear. **Methods:** A cross-sectional survey was administered to fourth-year nursing students from public and private universities. Instruments included the Health Professional’s Humanization Scale (HUMAS), the Communication Styles Inventory-Revised (CSI-R) and a sociodemographic questionnaire that captured prior training: completion of ≥6 h role-playing seminars in patient–family communication. **Results:** Mean scores were 3.62 ± 0.48 for HUMAS and 2.50 ± 0.52 for CSI-R. Women exceeded men on HUMAS total (*p* = 0.025) and on Sociability, Emotional Understanding, Dispositional Optimism and Self-Efficacy (all *p* ≤ 0.013), but not on Affect-Regulation or CSI-R. Age correlated weakly with Optimism (r = 0.24) and Self-Efficacy (r = 0.21). Students who had completed the role-playing seminars recorded higher HUMAS totals (d = 0.50; *p* = 0.001) and sub-scores, with only a modest gain in Affect-Regulation, and showed a trend towards better CSI-R performance (*p* = 0.06). No differences emerged by university type. HUMAS and CSI-R correlated moderately (r = 0.32; *p* = 0.001). In multivariate analysis, training (β = 0.36; *p* = 0.001) and CSI-R (β = 0.26; *p* = 0.001) jointly explained 27.9% of humanization variance; male sex exerted a small negative effect (β = −0.19; *p* = 0.001), whereas age was nonsignificant. **Conclusions:** Structured communication seminars are a key factor associated with higher levels of humanization in senior nursing students, whereas sociodemographic influences are modest. Embedding longitudinal, simulation-rich modules in communication and emotional intelligence is therefore recommended to cultivate truly person-centered nurses and to narrow observed sex disparities.

## 1. Introduction

Humanized care extends the traditional biomedical model by incorporating emotional, relational, and environmental dimensions into clinical practice [1]. In nursing, where patient contact is continuous, these competencies are associated with lower anxiety, improved treatment adherence, and higher patient satisfaction [2]. Humanization is currently understood as a set of professional skills aimed at safeguarding the patient’s overall well-being. This includes the integration of biological, psychological, and social dimensions, as well as the quality of relationships established with patients, families, and interdisciplinary teams [3,4]. These competencies have been operationalized into measurable domains such as dispositional optimism, sociability, emotional understanding, self-efficacy, and affect regulation, enabling their systematic evaluation and incorporation into educational programs.

Emotional intelligence plays a central role within this framework, as it enables clinicians to recognize, understand, and regulate their own emotions and those of others [5,6,7]. Higher levels of emotional intelligence have been associated with better clinical outcomes and lower occupational stress among nurses [8,9]. Similarly, empathy, defined as the ability to understand and, in some cases, share the patient’s emotional experience, is a key determinant of therapeutic relationships and care quality [10,11]. Evidence indicates that empathy can be enhanced through structured educational interventions [12] and is linked to reduced patient anxiety and improved outcomes in contexts such as oncology and palliative care [13]. Although age and sex may influence empathy expression [14], its development remains responsive to training due to neuroplasticity [15]. Studies using the Jefferson Scale of Empathy consistently report higher scores among women and a modest increase with age, suggesting the interaction of biological and sociocultural factors [16,17].

Therapeutic communication constitutes the primary mechanism through which emotional intelligence and empathy are expressed in clinical practice. Effective communication combines active listening with appropriate non-verbal behaviors and has been associated with lower professional stress and higher-quality care [18]. Students with stronger emotional competencies at the beginning of their training tend to acquire communication skills more efficiently, and structured educational approaches can further enhance this development [19,20]. Non-verbal communication, including posture, eye contact, vocal tone, and facial expression, conveys a substantial proportion of information during clinical interactions [18,21]. Mastery of these elements fosters trust and improves patient adherence [3,22]. However, many nursing students report anxiety and insecurity when interacting with patients, highlighting a gap between theoretical knowledge and clinical practice [2,23]. This gap supports the implementation of active learning strategies such as high-fidelity simulation, standardized-patient encounters, and virtual counseling tools [24,25,26,27,28,29,30].

The role of clinical faculty is also critical, as instructors who model humanized behaviors facilitate the acquisition of relational competencies in students [30,31]. In contrast, insufficient or fragmented training may hinder the development of communication, emotional intelligence, and empathy, negatively affecting both professional well-being and patient safety [2,7]. Targeted educational interventions have demonstrated measurable improvements in both humanization and communication skills [23,24,25,26].

Despite the growing recognition of humanization as a core component of nursing practice, empirical evidence regarding its prevalence and determinants among undergraduate students remains limited, particularly in the Spanish context. In response to this gap, the present cross-sectional study aims to: (i) quantify humanization and communication competence in senior nursing students, (ii) examine differences according to sex and age, (iii) assess the impact of structured communication training, and (iv) analyze the extent to which communication skills and sociodemographic variables predict levels of humanization.

## 2. Materials and Methods

### 2.1. Study Design and Participants

We carried out a cross-sectional, questionnaire-based investigation with non-probabilistic convenience sampling. This sampling strategy may limit the representativeness of the sample and the generalizability of the findings to the broader population of nursing students. Using non-probability convenience sampling, an invitation e-mail was distributed to fourth-year nursing students enrolled at two public and two private universities in central Spain. The message contained a secure Microsoft 365 Forms link that could be opened once from any device. To minimize the risk of duplicate responses, the survey platform was configured to allow a single submission per user account.

Inclusion criteria were: (a) age ≥ 18 years, (b) enrolment in clinical placements for ≥6 months, and (c) no prior professional nursing contract. To ensure the quality control of data, Records with more than 5% missing responses on either HUMAS or CSI-R were deleted listwise (n = 36; 11.5%). Records with more than 5% missing responses on either HUMAS or CSI-R were deleted using listwise deletion (n = 36; 11.5%). This approach was applied to preserve the internal consistency of composite scale scores, as incomplete responses could compromise reliability. Given the relatively low proportion of excluded cases, the impact on statistical power and representativeness was considered minimal.

No a priori sample size calculation was conducted, as the study followed an exploratory cross-sectional design. However, the final sample size (n = 277) was considered sufficient to detect small-to-moderate effect sizes in the main analyses.

### 2.2. Instruments

Data were collected with a three-part survey: (i) a sociodemographic questionnaire, (ii) the Health Professional’s Humanization Scale (HUMAS), and (iii) the Communication Styles Inventory-Revised (CSI-R).

The sociodemographic section included questions about age, sex, university type (public vs. private), and together with a dichotomous item on specialized training, defined as the completion of at least six hours of role-playing seminars dedicated to practicing and refining effective patient-communication skills. *“Have you completed role-playing seminars on effective communication with patients and their families led by clinical instructors?”* (0 = no; 1 = yes). The content and structure of these seminars were checked standardized across participating institutions, ensuring that all students received comparable training focused on patient–family communication through supervised role-playing activities.

The Health Professional’s Humanization Scale (HUMAS) [3] assesses five dimensions: Dispositional Optimism (OD), Sociability (SOC), Emotional Understanding (EU), Self-Efficacy (SE), defined as the ability to manage emotions and face difficult or stressful situations with confidence, and Affect Regulation (AF), understood as the capacity to modulate empathic engagement to avoid negative emotional consequences. The scale consists of items rated on a Likert-type scale, with higher scores indicating higher levels of humanization. Total and subscale scores are calculated as mean values, ranging from 1 to 5. All subscales demonstrated high internal consistency in the original validation study, with Cronbach’s alpha coefficients of 0.89 for Affect Regulation (AF), 0.86 for Emotional Understanding (EU), 0.88 for Self-Efficacy (SE), 0.85 for Dispositional Optimism (OD), and 0.86 for Sociability (SOC).

The CSI-R evaluates students’ ability to communicate effectively while respecting the interlocutor’s privacy [20]. It consists of 96 items rated on a five-point Likert scale (1 = strongly disagree; 5 = strongly agree), yielding total and dimensional scores ranging from 1 to 5. Higher scores reflect greater presence of the corresponding communication style traits. The instrument provides scores across six dimensions: Verbal Aggressiveness, Impression Manipulativeness, Questioningness, Expressiveness, Emotionality, and Preciseness. The inventory shows acceptable reliability (α = 0.81).

### 2.3. Statistical Analysis

Statistical analyses were performed with Jamovi 2.4.14. Normality and homoscedasticity tests were assessed as data-screening steps. Descriptives appear as mean ± SD or n (%). Group contrasts used independent-samples t tests when parametric assumptions held, Mann–Whitney U otherwise; categorical distributions were compared with χ^2^ and Cramér’s V. Associations between continuous scores employed Pearson or Spearman correlations, and effect sizes.

A separate hierarchical multiple-linear regression predicted HUMAS total: Block 1 = age, sex; Block 2 = university type, communication training; Block 3 = CSI-R total. Model assumptions, linearity, independence (Durbin–Watson), homoscedasticity (Breusch-Pagan), residual normality, multicollinearity (VIF < 5), were confirmed, and parameters are reported with standardized β, semi-partial η^2^, 95% bootstrap CIs (5000 resamples). Statistical significance was set at α = 0.05.

### 2.4. Ethical Considerations

The study protocol was approved by the Research Ethics Committee of Universidad Alfonso X el Sabio (2022_03/131) and complied with the principles of the Declaration of Helsinki and Regulation (EU) 2016/679 on personal data protection. Participation was voluntary and anonymous. Electronic informed consent was obtained from all participants before accessing the questionnaire.

This study was conducted and reported in accordance with the STROBE Statement for cross-sectional studies; the completed checklist (with page references) is provided as Supplementary File S1.

## 3. Results

### 3.1. Sample Description

After listwise deletion of incomplete cases (n = 36), the analytic sample comprised 277 fourth-year nursing students (62.5% women, 37.5% men). Mean age was 24.4 ± 1.8 years. A slight majority studied at public universities (55.2%), while 44.8% attended private institutions (Table 1). More than half of the students (55.9%) reported completing ≥ 6 h of role-playing seminars on effective patient–family communication. Average global scores were 3.62 ± 0.48 for humanization (HUMAS) and 2.50 ± 0.52 for communication competence (CSI-R). Among HUMAS domains, Dispositional Optimism ranked highest (3.75 ± 0.55) and Affect-Regulation lowest (3.50 ± 0.60) (Table 1).

### 3.2. Association Analysis Between Variables

Global HUMAS score increases modestly with age (r = 0.18, *p* = 0.006), driven mainly by Dispositional Optimism (r = 0.24, *p* = 0.001) and Self-Efficacy (r = 0.21, *p* = 0.001). No age-related pattern emerged for CSI-R. (Table 2).

As shown in Table 2, humanization was moderately correlated with communication competence (r = 0.32, *p* = 0.001). Age showed a weak positive correlation with HUMAS total (r = 0.18, *p* = 0.006), particularly with Dispositional Optimism and Self-Efficacy, while no significant association was observed between age and CSI-R.

Furthermore, in Table 3, women scored significantly higher than men on total HUMAS (t = 2.25, *p* = 0.025, d = 0.28; 95% CI [0.01, 0.21]), as well as on Sociability, Emotional Understanding, Dispositional Optimism, and Self-Efficacy (all *p* ≤ 0.013). No significant differences were observed for Affect Regulation (*p* = 0.50). Regarding communication competence, no statistically significant differences were found between sexes (t = 0.68, *p* = 0.50).

Students who had completed role-playing seminars in communication demonstrated significantly higher HUMAS total scores (t = 4.13, *p* = 0.001, d = 0.50), with consistent improvements across all subdimensions (all *p* < 0.001), except for Affect Regulation, where the effect was smaller (t = 2.34, *p* = 0.019, d = 0.28) (Table 3). For CSI-R, the difference between trained and untrained students approached statistical significance but did not reach statistical significance (t = 2.21, *p* = 0.06). No significant differences were observed according to type of university for either HUMAS or CSI-R scores, nor for any of their subdimensions (all *p* ≥ 0.05) (Table 3).

### 3.3. Predictive Model: Humanization

A hierarchical multiple regression was estimated with HUMAS total as the outcome (Table 4). After controlling for age and sex (block 1), adding university type and training (block 2) and finally CSI-R (block 3), the full model explained 27.9% of the variance (adjusted R^2^ = 0.265; F(4, 272) = 25.0, *p* = 0.001).

Specific training in communication emerged as the strongest positive predictor (β = 0.36). After controlling for the remaining variables, students who had completed the targeted workshop scored, on average, 0.236 HUMAS units higher than their untrained peers (B = 0.236; 95% CI 0.163 to 0.309; *p* = 0.001). Communication competence also displayed an independent, positive association with humanization (β = 0.258; B = 0.296; 95% CI 0.178 to 0.413; *p* = 0.001); each one-point increase on the CSI-R corresponded to an estimated 0.30-point rise in the HUMAS total. Sex retained a significant effect: being male was linked to a 0.13-point reduction in the outcome (β = −0.188; B = −0.134; 95% CI −0.215 to −0.053; *p* = 0.001) (Table 4).

Age showed only a marginal contribution (β = 0.087; B = 0.011; 95% CI –0.001 to 0.024; *p* = 0.075), suggesting that any age-related changes in humanization are small once sex, training and communicative ability are considered (Table 4).

## 4. Discussion

The present study provides evidence that humanization of care and communication competence are meaningfully interconnected constructs in senior nursing students. The positive association observed between HUMAS and CSI-R scores supports the conceptualization of therapeutic communication as a key behavioral channel through which emotional intelligence and empathy may be expressed in clinical practice [18]. This finding is consistent with prior literature indicating that both verbal and non-verbal communicative behaviors are essential for establishing trust, enhancing patient adherence, and improving overall care quality [3,22]. Even subtle non-verbal cues, such as a genuine smile, have been linked to better patient recovery trajectories, highlighting the practical relevance of relational competencies in clinical settings [22].

The level of humanization observed in this sample can be considered moderately high, aligning with findings reported in both practicing Spanish nurses [3] and international undergraduate cohorts [4]. Scores across most HUMAS dimensions suggest that, by the final year of training, students have developed a solid foundation in key humanistic competencies such as optimism, sociability, emotional understanding, and self-efficacy. However, the comparatively lower scores in Affect Regulation indicate a potential vulnerability in managing emotional involvement, a pattern also observed in early-career professionals where excessive empathic engagement may lead to emotional fatigue or burnout [14]. This imbalance suggests that while students can establish emotional connections with patients, they may lack sufficient strategies for regulating these interactions effectively.

Communication competence in this cohort appears to reflect an intermediate developmental stage. The CSI-R scores are comparable to those reported in validation studies among Spanish health-sciences students [20], suggesting that participants are neither highly skilled nor significantly deficient communicators. This transitional profile is consistent with longitudinal evidence describing the gradual acquisition of communication skills throughout nursing education [23]. Importantly, the moderate correlation between communication and humanization underscores the functional role of communication as a conduit for the expression of empathy and emotional intelligence in clinical contexts [18].

Differences observed by sex are consistent with well-established patterns in the literature. Female students scored higher on overall humanization and several of its dimensions, including sociability and emotional understanding, which aligns with studies linking gender socialization processes to caregiving orientations and empathic responsiveness [1,16,17]. Although these differences were statistically significant, their magnitude was small, suggesting that they should not be overinterpreted. Nevertheless, the persistence of a negative association for male sex in the regression model indicates that targeted educational strategies, such as role modeling, reflective training, or tailored communication exercises, may be beneficial in supporting the development of humanistic competencies across all student groups [32].

Age showed only modest associations with specific humanization dimensions, particularly optimism and self-efficacy, which is in line with developmental perspectives linking emotional regulation capacities to neurocognitive maturation [15,16]. However, its lack of significance in the multivariate model suggests that educational experiences, rather than age per se, may be more closely associated with levels of humanization in this population. This finding highlights the importance of structured training interventions in shaping professional competencies, even within relatively homogeneous age groups.

One of the most relevant findings of this study concerns the role of communication training. Participation in role-playing seminars was associated with higher levels of humanization, and consistent differences across most HUMAS dimensions. These results are in line with experimental studies demonstrating that simulation-based and experiential learning approaches can significantly enhance empathy, emotional intelligence, and prosocial behaviors in nursing students [20,23,24,25,26,27,28]. Notably, the relatively smaller differences observed in Affect Regulation suggest that this dimension may require more sustained or specialized interventions, such as mindfulness-based training or cognitive-emotional regulation techniques [14]. Given that only slightly more than half of the participants reported having received such training, these findings also point to a potential curricular gap that could be addressed through more systematic integration of communication modules. These findings should be interpreted with caution, as the cross-sectional design does not allow causal inference and the observed associations may be influenced by unmeasured factors.

Communication competence itself remained independently associated with humanization in the regression model, reinforcing its central role in patient-centered care. This association supports previous evidence indicating that relational skills are not merely complementary but foundational to effective nursing practice [20,28]. The literature further emphasizes the importance of non-verbal communication, such as eye contact, posture, and vocal tone, in shaping patient perceptions and clinical outcomes [23,28]. Emerging educational tools, including virtual simulation and AI-based training platforms, offer promising avenues for scaling these competencies while maintaining psychological safety and pedagogical efficiency [25,29].

From an educational perspective, these findings suggest that longitudinal and integrated approaches to communication training may be beneficial within nursing curricula. Embedding simulation-based learning, structured debriefing, and reflective practice across all academic years may be associated with the progressive consolidation of humanistic competencies. Additionally, aligning these training strategies with objective assessment methods, such as Objective Structured Clinical Examinations (OSCEs), could enhance both the validity of evaluation and the effectiveness of feedback processes [33,34]. Strengthening academic–practice partnerships may further support the transfer of these skills to clinical environments, for example through the incorporation of brief reflective practices or communication checklists during clinical placements.

Several limitations should be considered when interpreting these findings. First, the cross-sectional design precludes causal inference, limiting the ability to determine whether communication competence leads to greater humanization or vice versa. Second, the use of non-probabilistic convenience sampling may introduce selection bias and restrict the generalizability of results beyond the participating institutions; in particular, students with a greater interest in humanization or communication may have been more likely to participate, potentially leading to an overestimation of the observed levels of these competencies. Third, reliance on self-report instruments introduces the possibility of self-report bias, including common-method variance and social desirability bias, particularly in constructs such as empathy and humanization, which are socially valued in healthcare contexts. Participants may have overestimated their competencies, potentially inflating observed associations. Finally, the sample was restricted to final-year nursing students, which limits the extrapolation of results to earlier stages of training or to practicing professionals.

Future research using longitudinal or experimental designs is needed to better understand the directionality and potential causal mechanisms underlying the relationship between communication competence and humanization.

## 5. Conclusions

This study confirms that Senior nursing students in four Spanish universities displayed intermediate-to-high levels of humanized-care competence and moderate communication proficiency. A clearly delimited, communication training on patient and family-centered communication emerged as the most powerful modifiable determinant of humanization, being associated with higher scores in four of the five HUMAS domains and accounting, together with overall communication ability, for almost 28% of the total variance. Sex exerted a secondary, unfavorable effect for male students, whereas age and university ownership were negligible once education-related variables were controlled.

These results demonstrate that humanization is shaped less by immutable personal traits than by structured experiential learning that integrates theory, simulation and guided reflection. Nursing faculties should therefore embed mandatory, longitudinal modules in therapeutic communication and emotional intelligence across all academic years, regularly monitor outcomes with validated tools such as HUMAS and CSI-R, and provide targeted support for male students and for the under-developed affect-regulation domain. Future longitudinal or experimental designs, triangulating self-report scales with behavioral and patient-reported measures, are warranted to confirm causality and track the durability of training effects into professional practice.

## Figures and Tables

**Table 1:** Description of the sample.

Variable	Level	n (%)	Mean	SD	Median	Min	Max	95% CI
Sex	Female	173 (62.45)	-	-	-	-	-	-
	Male	104 (37.55)	-	-	-	-	-	-
Type of University	Public	153 (55.23)	-	-	-	-	-	-
	Private	124 (44.77)	-	-	-	-	-	-
Specific training in communication	Yes	155 (55.96)	-	-	-	-	-	-
	No	122 (44.04)	-	-	-	-	-	-
Age (years)	-	277	24.4	1.8	24	21	26	24.19–24.61
HUMAS total	-	277	3.62	0.48	3.68	2.41	4.74	3.56–3.73
OD	-	277	3.75	0.55	3.86	2.31	4.86	3.63–3.87
SOC	-	277	3.55	0.62	3.54	2.00	4.89	3.40–3.70
EU	-	277	3.68	0.50	3.73	2.47	4.80	3.56–3.79
SE	-	277	3.70	0.58	3.79	2.21	4.84	3.56–3.84
AF	-	277	3.50	0.60	3.49	2.08	4.83	3.35–3.65
CSI-R total	-	277	2.50	0.52	2.48	1.31	3.76	2.38–2.61

**Table 2:** Correlations between age, humanization (HUMAS), and communication competence (CSI-R).

Variable	Age	HUMAS Total	CSI-R Total
Age	-	0.18 **	−0.07
HUMAS total	0.18 **	-	0.32 ***
CSI-R total	−0.07	0.32 ***	-

Note: Pearson correlation coefficients are reported. ** *p* < 0.01; *** *p* < 0.001.

**Table 3:** Differences in humanization (HUMAS) and communication competence (CSI-R) according to sex, communication training, and type of university.

Variable	Group	HUMAS		CSI-R			
		Mean ± SD	95%CI	Mean ± SD	95%CI	t; *p*-Value	Cohen’s d
Sex	Female	3.51 ± 0.37	[3.45, 3.56]	2.42 ± 0.54	[2.34, 2.50]	t = 2.25; *p* = 0.025	0.28
	Male	3.40 ± 0.48	[3.31, 3.49]	2.67 ± 0.49	[2.57, 2.76]		
Communication training	Yes	3.55 ± 0.35	[3.49, 3.60]	2.43 ± 0.55	[2.34, 2.52]	t = 4.13; *p* = 0.001	0.5
	No	3.35 ± 0.46	[3.27, 3.43]	2.59 ± 0.49	[2.50, 2.68]		
Type of university	Public	3.47 ± 0.40	[3.41, 3.53]	2.49 ± 0.55	[2.40, 2.58]	t = 0.17; *p* = 0.868	0.02
	Private	3.50 ± 0.41	[3.43, 3.57]	2.47 ± 0.52	[2.38, 2.56]		

**Table 4:** Multiple linear regression predicting total HUMAS score.

Predictor	B	SE B	β	t	*p*	95% CI B	VIF
Constant	1.97	0.31	-	6.28	=0.001	1.36–2.57	-
Age (years)	0.011	0.006	0.087	1.79	0.075	−0.001–0.024	1.05
Sex (0 = F, 1 = M)	−0.134	0.041	−0.188	−3.25	0.001	−0.215–0.053	1.07
Training in communication (0 = no, 1 = yes)	0.236	0.037	0.358	6.39	=0.001	0.163–0.309	1.08
CSI-R total	0.296	0.060	0.258	4.92	=0.001	0.178–0.413	1.12

Note: Model summary: R^2^ = 0.279; adjusted R^2^ = 0.265; F(4, 272) = 25.0; *p* = 0.001. Regression assumptions were satisfied (Durbin–Watson = 1.98; Breusch–Pagan *p* = 0.40; VIF range 1.05–1.12).

## Data Availability

The datasets presented in this article are not readily available because the data are part of an ongoing study. In the meantime, additional materials and specific data supporting the findings of this study can be made available upon reasonable request to the corresponding author.

## Supplementary Materials

The following supporting information can be downloaded at: https://www.mdpi.com/article/10.3390/nursrep16050171/s1, File S1: STROBE Statement—Checklist of items that should be included in reports of cross-sectional studies.

## Public Involvement Statement

No public involvement in any aspect of this research.

## Guidelines and Standards Statement

This manuscript was prepared in accordance with the STROBE (Strengthening the Reporting of Observational Studies in Epidemiology) guidelines for observational research.
