# Supplementary material for: Humanization and Communication Skills: A Cross-Sectional Study in Spanish Nursing Students
Source: Nurs Rep. 2026 May 18;16(5):171. doi: 10.3390/nursrep16050171 (PMC13209899; doi:10.3390/nursrep16050171)
Supplement: Supplementary file 1 [file nursrep-16-00171-s001.zip › nursrep-4245678-supplementary.pdf]

STROBE Statement—Checklist of items that should be included in reports of cross-sectional studies

Manuscript title: Humanization and Communication Skills: A Cross-Sectional Study in Spanish Nursing Students

Item No	Recommendation	Reported on page No	Where in the manuscript this is addressed
1	Indicate the study's design with a commonly used term in the title or the abstract	Throughout manuscript	Title includes 'A Cross-Sectional Study'; abstract states it was a cross-sectional survey.
2	Provide in the abstract an informative and balanced summary of what was done and what was found	Throughout manuscript	Abstract clearly describes objectives, methods, key results, and conclusions.
3	Explain the scientific background and rationale for the investigation being reported	Throughout manuscript	Introduction discusses humanization, empathy, and communication in nursing context with references.
4	State specific objectives, including any prespecified hypotheses	Throughout manuscript	Objectives are listed at the end of Introduction (four defined objectives).
5	Present key elements of study design early in the paper	Throughout manuscript	Design described in section 2.1 as cross-sectional, questionnaire-based.

6	Describe the setting, locations, and relevant dates, including periods of recruitment and data collection	Throughout manuscript	Data collected from four Spanish universities; no specific dates provided, but context described.
7	Give the eligibility criteria, and the sources and methods of selection of participants	Throughout manuscript	Eligibility criteria in 2.1: age ≥18, final-year nursing students, ≥6 months placements, no prior contracts.
8	Clearly define all outcomes, exposures, predictors, potential confounders, and effect modifiers. Give diagnostic criteria, if applicable	Throughout manuscript	Outcomes: HUMAS and CSI-R total scores; predictors: sex, age, training, university type; regression model explained.
9	For each variable of interest, give sources of data and details of methods of assessment (measurement). Describe comparability of assessment methods if there is more than one group	Throughout manuscript	Measurement instruments (HUMAS, CSI-R) described in detail in section 2.2 with reliability values.
10	Describe any efforts to address potential sources of bias	Throughout manuscript	Data quality control: records with >5% missing data were deleted; ethical oversight ensured anonymity.
11	Explain how the study size was	Throughout	Final sample after listwise deletion

	arrived at	manuscript	was 277; sampling method was convenience-based.
12	Explain how quantitative variables were handled in the analyses. If applicable, describe which groupings were chosen and why	Throughout manuscript	Continuous variables analyzed as means ± SD; categorical variables dichotomized (e.g., training yes/no).
13	Describe all statistical methods, including those used to control for confounding	Throughout manuscript	Statistical analyses (t tests, correlations, regression) described in 2.3; regression included confounders.
14	Report numbers of individuals at each stage of study—e.g., numbers potentially eligible, examined for eligibility, included in the study, and analyzed	Throughout manuscript	Described in 3.1: 313 recruited, 36 removed, 277 analyzed.
15	Give characteristics of study participants (e.g., demographic, clinical, social) and information on exposures and potential confounders	Throughout manuscript	Table 1 shows sex, age, university type, training status, and main scores.
16	Report numbers of outcome events or summary measures	Throughout manuscript	Mean and SD for HUMAS, CSI-R, and subscales reported in Table 1.

17	Give unadjusted estimates and, if applicable, confounder-adjusted estimates and their precision (e.g., 95% CI). Make clear which confounders were adjusted for and why they were included	Throughout manuscript	Regression Table 3 reports standardized and unstandardized coefficients, CIs, and adjusted R^2.
18	Report category boundaries when continuous variables were categorized	Throughout manuscript	Training dichotomized as Yes/No; sex as Female/Male; age continuous.
19	Report other analyses done—e.g., analyses of subgroups and interactions, and sensitivity analyses	Throughout manuscript	Subgroup comparisons by sex, age, and training; interaction terms not included.
20	Summarize key results with reference to study objectives	Throughout manuscript	Section 4 opens summarizing results relative to four objectives.
21	Discuss limitations of the study, taking into account sources of potential bias or imprecision	Throughout manuscript	Limitations listed: cross-sectional design, non-probabilistic sampling, and final-year-only sample.
22	Give a cautious overall interpretation of results considering objectives, limitations, multiplicity of	Throughout manuscript	Interpretation integrates with prior literature on empathy, communication, and training.

	analyses, results from similar studies, and other relevant evidence		
23	Discuss the generalisability (external validity) of the study results	Throughout manuscript	Generalizability limited to Spanish senior nursing students; noted in Discussion.
24	Give the source of funding and the role of the funders for the present study and, if applicable, for the original study on which the present article is based	Throughout manuscript	Funding section states no specific grant; competing interests declared none.
